# A Rare Case of Gemcitabine-Induced Pulmonary Hypertension

**DOI:** 10.17140/PRRMOJ-5-139

**Published:** 2019-12-20

**Authors:** Janice Shen, Su Yun Chung, Elham Azimi-Nekoo, Jyothi Jose, Muhammad W. Saif

**Affiliations:** 1 Division of Hematology-Oncology, Department of Medicine, Northwell Health, Manhasset, NY, USA; 2 Northwell Health Cancer Institute, Monter Cancer Center, Lake Success, NY, USA; 3 Donald and Barbara Zucker School of Medicine at Hofstra/Northwell, Hempstead, NY, USA

**Keywords:** Cholangiocarcinoma, Gemcitabine, Pulmonary hypertension

## Abstract

**Context:**

Gemcitabine is the backbone of systemic treatment of locally advanced and metastatic intrahepatic cholangiocarcinoma. In recent literature, gemcitabine has been linked to various pulmonary side effects.

**Case Report:**

We report a case of an 82-year-old male who developed acute pulmonary hypertension after receiving one cycle of gemcitabine for metastatic cholangiocarcinoma. His symptoms began with fatigue associated with shortness of breath and cough that worsened despite dose reduction. He developed new onset bilateral pulmonary effusions and an echocardiogram revealed findings consistent with pulmonary hypertension. A computed tomography (CT) angiogram was negative for pulmonary thromboembolism. Although he was promptly treated with diuretics and steroids, the patient could not tolerate any further therapy.

**Conclusion:**

Gemcitabine-induced pulmonary hypertension is rare and can be challenging to diagnose, as it remains a diagnosis of exclusion. However, physicians should be vigilant of new pulmonary symptoms, as delayed treatment can cause significant patient morbidity and mortality.

## INTRODUCTION

Intrahepatic cholangiocarcinoma is a rare yet aggressive cancer of the biliary tract that portends a poor prognosis.^1^ A majority of patients are diagnosed when their disease has already reached a non-resectable, advanced state with a five-year survival of only 30%.^2^ Systemic therapy with gemcitabine is often used as either a single agent or in combination with other chemotherapy drugs for both locally advanced and metastatic disease. Gemcitabine is generally well-tolerated, and its most common adverse effects are myelosuppression and gastrointestinal toxicities. In recent literature, gemcitabine has been linked to a variety of severe pulmonary side effects. Despite its low incidence, the spectrum of pulmonary injury is wide, including potentially fatal conditions.^3^ We report a case of acute pulmonary hypertension in a patient treated with gemcitabine for metastatic intrahepatic cholangiocarcinoma.

## CASE REPORT

An 82-year-old man was brought to the hospital after sustaining a mechanical fall at home and was found to have a non-operable left greater trochanter fracture. On abdominal imaging, he was incidentally found to have a 9.2 cm dominant mass at the dome of the liver straddling the left and right hepatic lobes with a 6 mm right lower lobe pulmonary nodule, which was suspicious for metastatic intrahepatic cholangiocarcinoma. A subsequent liver biopsy confirmed moderately differentiated adenocarcinoma. Based on his age and performance status, the patient began treatment with single agent gemcitabine 1000 mg/m^2^ on days one and eight every three weeks in conjunction with pegfilgrastim.

**Figure 1.**
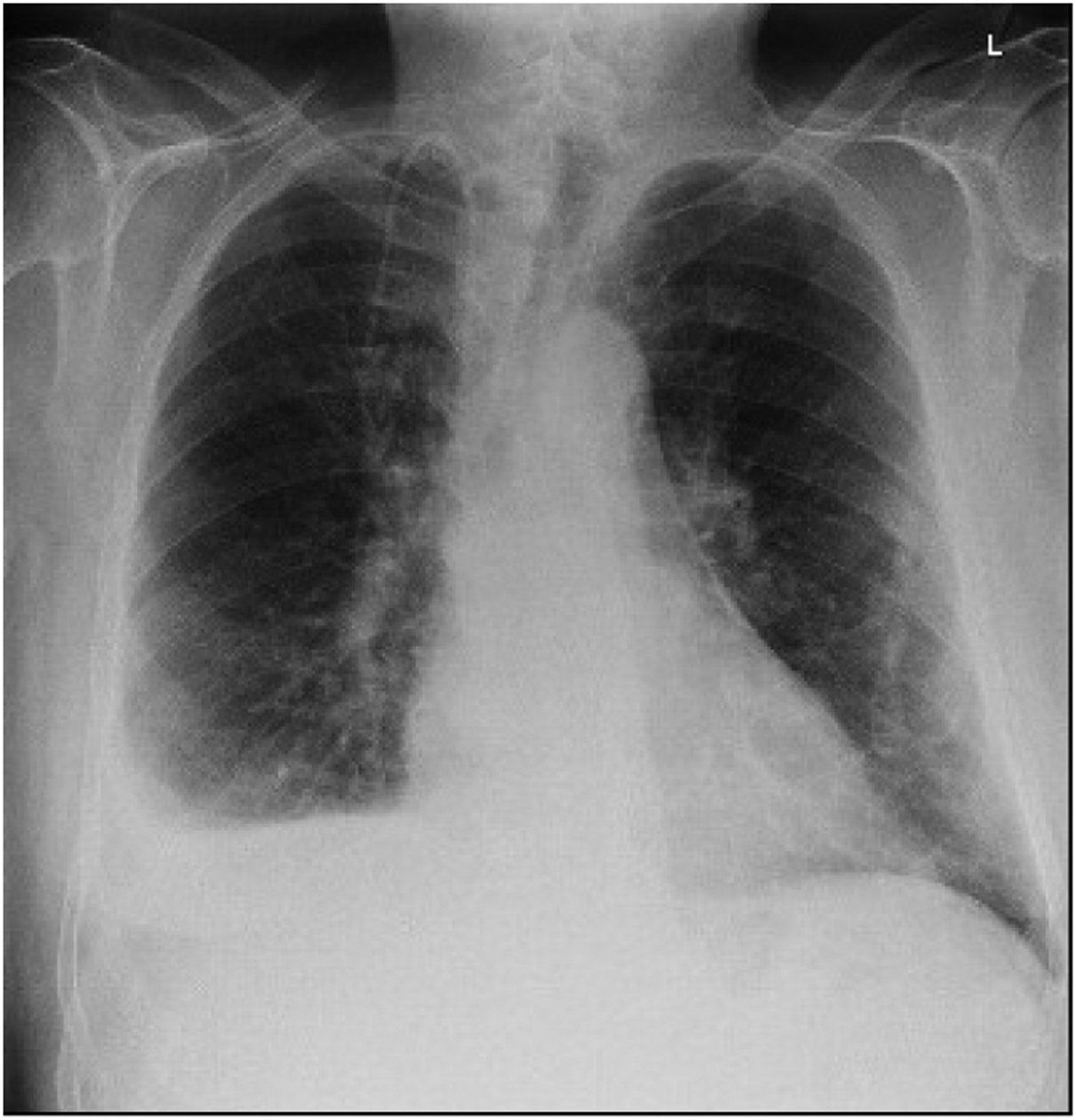
Development of a Right Pleural Effusion after Gemcitabine Treatment

**Figure 2.**
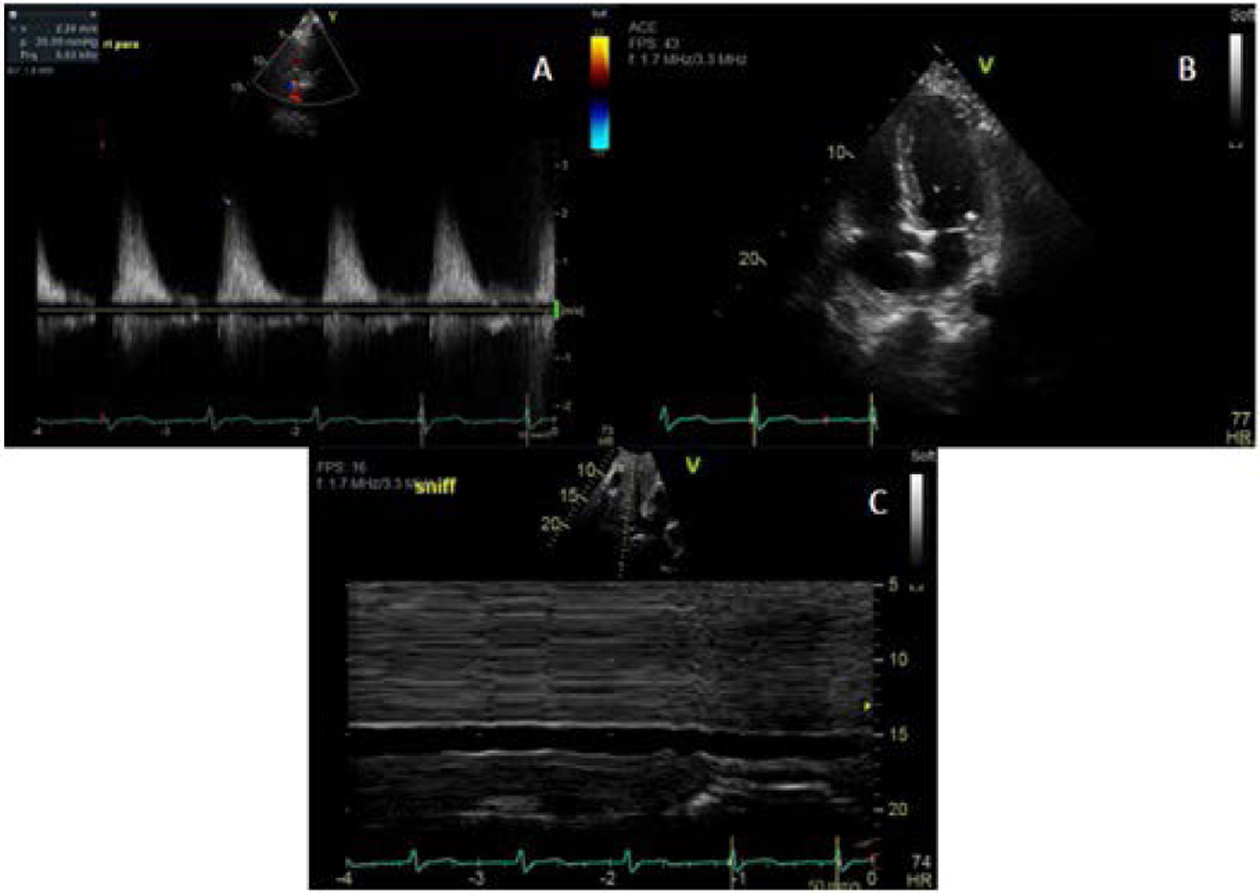
Images A-C Showing Echocardiographic Evidence of Pulmonary Hypertension

On the fourth day of his first cycle with gemcitabine, the patient developed a blanchable maculopapular rash on his upper chest, which eventually resolved with loratadine and diphenhydramine. However, the patient became increasingly fatigued after day eight of gemcitabine. Due to these side effects, gemcitabine was dose reduced to 500 mg/m^2^ every other week. Although his fatigue improved on the days he did not receive chemotherapy, the patient complained of new onset of shortness of breath and a dry cough that persisted into his second treatment cycle. During an office visit, a pulse oximetry measurement registered the patient as breathing 92% on ambient air. A chest X-ray showed bilateral pleural effusions (Figure 1). Even after initiating furosemide to facilitate diuresis and a short course of corticosteroids, the patient continued to have dyspnea on exertion. The patient, who had no significant history of cardiac or pulmonary disease, underwent a transthoracic echocardiogram, which revealed an estimated pulmonary artery systolic pressure of 35 mmHg assuming a right atrial pressure of 15 mmHg; this finding was consistent with pulmonary hypertension likely secondary to gemcitabine (Figure 2). Computed tomography (CT) angiogram of the chest was performed to rule out pulmonary thromboembolism (PE), and it was negative. The patient was promptly treated with diuretics and gemcitabine was discontinued given the high suspicion of drug related toxicity causing pulmonary hypertension.

The patient could not tolerate any further treatment with gemcitabine at which point his regimen was changed to fluorouracil and leucovorin. Despite this, he endured worsening symptoms and ultimately opted for hospice care.

## DISCUSSION

Gemcitabine is a pyrimidine analog that is used to treat several malignancies including biliary tract cancers, pancreatic cancer, non-small cell lung cancer, and breast cancer.^4^ Although it is relatively well tolerated despite its tendency to be myelosuppressive, gemcitabine-induced pulmonary toxicity (GIPT) is a rare yet critical entity whose incidence remains unknown. Various types of lung injuries have been reported with gemcitabine use including interstitial pneumonitis, diffuse alveolar damage, pulmonary fibrosis, and acute respiratory distress syndrome.^5,6^

While the exact mechanism is unknown, several hypotheses have been proposed to explain the pathogenesis of GIPT. For example, the induction of pro-inflammatory cytokines and an enhanced expression of growth factors are linked to idiopathic pulmonary fibrosis related to gemcitabine use. Furthermore, causative mechanisms including damage to alveoli, pulmonary vasculature, and/or the interstitium may also explain the development of pulmonary hypertension in these patients. In one animal study performed to assess acute and delayed toxicities of gemcitabine, it was found that the drug induces vasoconstriction of the pulmonary capillaries, causing increased mean left main pulmonary arterial pressure.^7^

Pulmonary hypertension, diagnosed through right heart catheterization, is defined by a mean pulmonary artery pressure ≥25 mmHg at rest.^8^ In our case, the patient was found to have a pulmonary artery pressure of 35 mmHg. Therefore, right heart catheterization was not performed because clinical suspicion was high enough to start treatment quickly based on the patient’s symptoms and diagnostic transthoracic echocardiogram findings in the absence of PE. Despite rapid initiation of treatment with diuretics and steroids, he still only had a suboptimal response likely due to coexisting pulmonary conditions, such as worsening metastatic nodules and malignant pulmonary effusion, which developed later in his disease course.

The time frame from gemcitabine initiation to development of pulmonary toxicity widely varies from as early as 3-days to as late as one year.^5^ This makes it challenging for physicians to diagnose GIPT and begin treatment especially when the exact underlying mechanism remains unknown. Treatment options also vary depending upon the type of lung toxicity. Multiple etiologies such as pulmonary vaso-occlusion, capillary leak, and cytokine-mediated direct toxicity can all contribute to gemcitabine-induced pulmonary hypertension.^5,9^ For GIPT, conventional modalities such as diuretics and steroids have been used, however, their benefit in pulmonary hypertension is questionable.^4,10,11^ Typically, drug-related pulmonary arterial hypertension is treated with prostacyclin analogues and calcium channel blockers, however, further studies are needed to assess their efficacy in cases associated with gemcitabine. Perhaps anti-cytokine agents could also be implemented to reduce the cytokine burden incited by gemcitabine.

## CONCLUSION

Gemcitabine-induced pulmonary hypertension and other lung toxicities remain a diagnosis of exclusion. However, physicians should remain vigilant of detecting and treating symptoms when they arise. Untreated pulmonary hypertension can cause significant morbidity and mortality, and therefore, early recognition of this condition is essential. Further development of new treatment modalities, based on the suspected mechanisms, is needed to ensure good patient outcomes.

## Abbreviations

CT: Computed Tomography
PE: Pulmonary Thromboembolism
GIPT: Gemcitabine-Induced Pulmonary Toxicity
